# Supplementation of hairy eggplant (*Solanum ferox*) and bitter ginger (*Zingiber zerumbet*) extracts as phytobiotic agents on whiteleg shrimp (*Litopenaeus vannamei*)

**DOI:** 10.5455/javar.2022.i571

**Published:** 2022-02-05

**Authors:** Sinung Rahardjo, Merary A. The Vauza, Djumbuh Rukmono, Putu Angga Wiradana

**Affiliations:** 1 Department of Aquaculture, Faculty of Utilization Fisheries, Jakarta Fisheries University, Jakarta, Indonesia; 2 Master Student of Fisheries Resource Utilization, Postgraduate Program of Jakarta Fisheries University, Jakarta, Indonesia; 3 Study Program of Biology, Faculty of Health, Science, and Technology, Universitas Dhyana Pura, Provinsi Bali, Indonesia

**Keywords:** Growth, hemocytes, phytobiotics agent, shrimp *Litopenaeus vannamei*

## Abstract

**Objective:**

This study aimed to evaluate the combination of hairy eggplant *(Solanum ferox)* and bitter ginger *(Zingiber zerumbet)* on the production performance and hematological parameters of whiteleg shrimp *(Litopenaeus vannamei).*

**Materials and Methods:**

Four treatments were formulated in the test feed, where P1 (control + commercial vitamin C); P2 (120 ml/l combination of *S. ferox* and *Z. zerumbet*); P3 (100 ml/l); and P4 (80 ml/l) 6,000 post-larvae shrimp with an average initial weight of 0.2 gm were randomly stocked in four groups, with three replications per treatment, and 500 were stocked in each pond with a total of 12 pounds.

**Results:**

Based on the results, there were significant differences in production performance (survival, absolute weight growth, specific growth rate, and feed conversion ratio). Biologically, the best performance was found in the P3 treatment (100 ml/l). In this treatment, the total number of hemocytes and the number of hyaline hemocytes were much higher, and this was not the case in the control treatment (P1), where the number of semi-granular and granular cells was significantly higher than the treatment group *(p* < *0.05)*.

**Conclusions:**

This study confirmed that supplementation of 100 ml/l of *S. ferox* and *Z. zerumbet* could improve the production performance and hemato-immunological parameters of whiteleg shrimp, with functional potential to be developed in phytobiotic-based commercial diets for shrimp.

## Introduction

Crustacean production has increased to 6.09 million tons, or equivalent to USD 36.2 million, due to increased consumption of shrimp commodities worldwide [1]. Whiteleg shrimp (*Litopenaeus vannamei*) are one of the most popular aquaculture commodities and are relatively easy to cultivate [2]. This can be proven by the high percentage of Pacific whiteleg shrimp cultivation worldwide, at around 90%. This shrimp can adapt to a broad salinity range (euryhaline) between 5 and 30 parts per trillion; cultivate with a high stocking density; and grow sufficiently with low protein feeds, making this shrimp a leading commodity in Indonesia.

However, an increase in shrimp production with unmatched water quality management, quality seeds, or superior broodstock to quality feed can harm shrimp productivity by causing various diseases. Several disease outbreaks in shrimp ponds are related to viral diseases like the white spot syndrome virus and Taura syndrome virus. On the contrary, bacterial diseases like *Vibrio harveyi*, *V. alginolyticus*, and *V. parahemolyticus* can cause considerable losses to shrimp hatcheries worldwide [3].

The whiteleg shrimp’s immune systems are also essential for the emergence of a disease in the aquaculture environment. Crustaceans, especially shrimp, only have a nonspecific and humoral natural immune system [4]. This causes the growth and immunity of shrimp to be increased, depending on the quality of the broodstock, the environment, and the application of biocontrol genes [5,6].

Currently, antibiotics and chemotherapy agents are still used to control bacterial diseases [7,8]. However, the use of antibiotics unwisely can lead to the accumulation of residues in the tissue, resulting in a decrease in product quality, the emergence of antibiotic resistance problems in shrimp and other aquatic organisms [9], and pollution of the aquatic environment [10]. Therefore, the use of environmentally friendly materials is needed to control diseases in the current aquaculture system. Several approaches, such as the use of pro-, pre-, and synbiotics [11], immunostimulants [12], vaccination [13], quorum-sensing, phage application [14], RNA interference [15], development of molecular-based diagnostic materials, and the breeding and spawning of specific pathogen-free shrimp [16], have attracted the attention of researchers and shrimp farmers.Phytobiotics are functional additives derived from nature, including food supplement ingredients that have become an alternative to improve health and resistance to disease attacks that are ecologically correct in the modern aquaculture sector [17]. The development of phytobiotic materials is the best effort to improve the shrimp immune system. In addition to being compatible with the shrimp immune system, which is still primitive (nonspecific immunity), phytobiotics also have several advantages, such as abundant sources of materials, a wide target range, the potential for large-scale application, and environmental friendliness.

Some of the natural agents that can be used as phytobiotics to increase the immune system of whiteleg shrimp are hairy eggplant (*Solanum ferox*) and bitter ginger (*Zingiber zerumbet*) [18]. Hairy eggplant, or *S. ferox*, is widely planted in tropical areas such as Indonesia for its fruit. This plant has been listed as a medicinal plant in the ethnobotanical inventory because it is reported to be effective in treating human diseases. Hairy eggplants contain bioactive compounds such as phenolics, flavonoids, and polyphenols that play an essential role in preventing oxidative stress and several biological effects such as antioxidants and anti-inflammatory [19]. Another traditional plant such as *Z. zerumbet* (L), part of the *Zingiberaceae* family, is commonly found in Southeast Asia, such as Indonesia. This plant is traditionally used in various cuisines and drinks because it is reported to have anti-allergic properties in its rhizomes.

Previous studies have shown that the combination of *Z. zerumbet* and *Curcuma zedoria* added to the orange-spotted grouper (*Epinephelus coioides*) was able to act as an immunostimulant by increasing nonspecific immune responses (respiratory burst activity, reactive oxygen species, phagocytic activity, superoxide dismustase, and lysozyme activity) [20]. Hardi et al. [21] explained that the combination of *Boesenbergia pandurate*, *Z. zerumbet*, and *S. ferox* effectively prevents infection with *Aeromonas hydrophila* and *Pseudomonas* sp., and modulates the immune system of tilapia, *Oreochromis niloticus*. However, studies highlighting the potential of phytobiotics have not been reported in white shrimp, although compound derivatives of phytobiotics have been previously reported [22].

Based on this report, this study aimed to evaluate the combination of hairy eggplant (*S. ferox*) and bitter ginger (*Z. zerumbet*) in increasing the production performance and immune system of whiteleg shrimp (*L. vannamei*). This study is expected to provide valuable preliminary information for applying environmentally friendly immunostimulants to increase Pacific white shrimp production.

## Materials and Methods

### Ethical approval

This research has been licensed by the Fisheries Expert Polytechnic (AUP), Marine and Fisheries Research and Human Resources Agency (BRSDM), Ministry of Marine Affairs and Fisheries (KKP) of the Republic of Indonesia with No: 1832/POLTEK-AUP/TU.210/IX/2020.

### Study area and period

This study was conducted from September 2020 to December 2020 at the Loka Pengelolaan Sumber Daya Pesisir and Laut Serang, Directorate General of Marine Space Management, Ministry of Marine and Fisheries, Republic of Indonesia.

### Study design

This study used a completely randomized design with four treatments and three replications. The treatments used were a combination of hairy eggplant (*S. ferox*) and bitter ginger (*Z. zerumbet*) with the following details:

P1 : no combination + vitamin C (control);

P2: the combination of *S. ferox* and *Z. zerumbet* with a dose of 120 ml/l;

P3: the combination of *S. ferox* and *Z. zerumbet* with a dose of 100 ml/l;

P4: the combination of *S. ferox* and *Z. zerumbet* with a dose of 80 ml/l.

### Preparation of S. ferox and Z. zerumbet

For preparation of the extract, we referred to the study by Hardi et al. [21]. In short, the plants were collected from traditional markets. The materials were washed and dried in an oven at 40°C–45°C for 48 h. The dry ingredients were then ground using a blender to form a fine powder. Exactly 100 gm of dry samples were mixed with 100 ml of 96% ethanol in an Erlenmeyer flask at room temperature for 72 h. The mixture was separated using 0.5 m of Whatman filter paper to obtain the extract filtrate. The filtrate was re-evaporated using a rotary evaporator to separate the ethanol content for 3–5 h. The extract was stored in the refrigerator for further testing.

### Rearing of whiteleg shrimp

**Figure 1.**
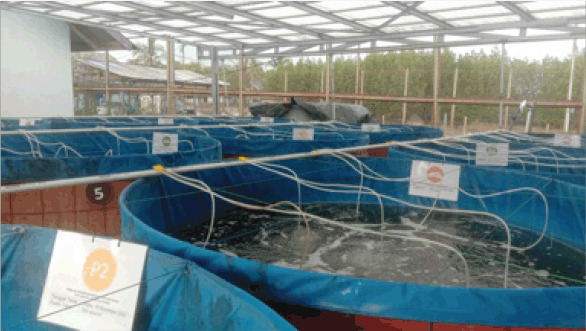
Rearing pond of *L. vannamei* in this study.

Rearing of whiteleg shrimp was carried out in a round tarpaulin container with a pool diameter of 2 m and a height of 1 m, for a total of 12 pools. Each pond has nine aeration points evenly distributed in the rearing pond (Fig. 1). The extract was mixed into the feed and the dose was adjusted for each treatment. Feeding was carried out four times a day at 07.00 a.m., 11.00 a.m., 3.00 p.m., and 7.00 p.m., respectively. The amount of feed given was adjusted to the feeding rate according to the Standard National Indonesia No. 01-7246-2006, namely 15%–10%.

### Production performance parameters

#### Survival rate (SR)

The SR of whiteleg shrimp was calculated at the end of the rearing period. The calculation was carried out according to the equations described in Wiradana et al. [23] and Fendjalang et al. [24] as follows:


$$\mathrm{SR}\mathrm{(\%)}=\frac{\mathrm{Nt}}{\mathrm{No}}\times 100\%$$

Where

SR: Survival rate (%);

*Nt:* Number of shrimp at the end of the study (individual);

*No:* Number of shrimp at the beginning of the study (individual).

#### Absolute weight growth

Weight growth was observed every 7 days by weighing the number of shrimp. Absolute weight growth based on Widanarni et al. [25] is as follows:

Δ*W* =*Wt* − *Wo*

Where

Δ*W*: Absolute weight growth (gm);

*Wt*: Final shrimp weight (gm);

*Wo*: Initial shrimp weight (gm).

#### Specific growth rate (SGR) 

The SGR was observed once a week based on Zubaidah et al. [26] as follows:


$$\mathrm{SGR}\mathrm{(\%/}\mathrm{day})=\frac{\mathrm{LnWt}-\mathrm{LnWo}}{T}\times 100\%$$

Note:

SGR(%/day): Specific growth rate;

*Wt*: Final shrimp biomass (gm);

*Wo*: Initial shrimp biomass (gm);

*T* : Rearing period (days).

#### Feed conversion ratio (FCR) 

FCR was calculated at the end of the rearing period based on Sarjito et al. [27] as follows:


$$\mathrm{FCR}=\frac{\sum F\;\mathrm{feed}\;\mathrm{given}-\sum F\;\mathrm{leftover}\;\mathrm{feed}}{(\mathrm{Bt}+\mathrm{Bm})-\mathrm{Bo}}$$

Where

FCR: Feed conversion ratio (gm);

Σ*F*: Feeding amount (gm);

*Bt*: Final shrimp biomass (gm);

*Bm*: Dead shrimp biomass (gm);

*Bo*: Initial shrimp biomass (gm).

#### Water quality

To maintain the media quality, daily draining of water was carried out [28]. Water quality parameters measured include temperature, dissolved oxygen (DO), pH, ammonia, nitrate, and nitrite. Water quality parameters are used as supporting data in this study.

#### Immunity parameters

The whiteleg shrimp immune response was evaluated by calculating the total hemocyte count (THC) and differential hemocyte counts (DHC). Collection and preparation of shrimp hemolymph were carried out by Zahra et al. [28]. THC and DHC were analyzed by taking 0.1 ml of hemolymph in the fifth pereopod using a 1 ml syringe filled with 0.3 ml of Na-EDTA anticoagulant to prevent blood clots. The sample was homogenized for 5 min in a moistened microtube with 10% Na-EDTA. Hemolymph was dripped on the hemocytometer and closed using a cover glass. Calculation of the number and type of cells was carried out under a light microscope with a magnification of 400×. Observation of immune parameters was carried out at the end of the rearing period (56 days). The calculation of THC and DHC refers to the method by Suleman et al. [29] as follows:


$$\mathrm{DHC}\mathrm{(\%)}=\frac{\sum A}{\sum B}\times 100$$

Note:

Σ*A:* The number of each hemocyte cell type;

Σ*B:* THC.

### Histological analysis

The gill tissue was placed in Davidson’s solution until dehydrated, embedded in paraffin, and sliced using a microtome. The tissue was stained using hematoxylin and eosin and analyzed to identify defects such as hyperplasia, vacuolation, and necrosis. The histology is shown in the figures defined by Mari et al. [30].

### Data analysis

Production performance parameters and immunity were tabulated using MS Excel 2019 (Microsoft, USA). Statistical analysis was carried out using Statistical Package for the Social Sciences Version 25 software (IBM, USA). The data underwent a homogenicity test, followed by a single-factor analysis of variance with a 95% confidence interval. To find out the differences in each treatment, the data underwent Duncan’s test. The statistical results were interpreted and presented in the form of tables and figures.

## Results and Discussion

### Production performance

The supplementation of hairy eggplant and bitter ginger in feed can increase absolute weight growth in whiteleg shrimp. The highest absolute weight growth was 9.19 gm/individual and was found in P3, then in P4 (6.97 gm/individual), P2 (6.03 gm/individual), and P1 (control) (5.82 gm/individual) (Table 1). Weight gain can occur from energy entering the body. The energy obtained from feed ingredients will first be used for rearing activities, then the remaining energy will be used for the growth process [31,32]. The absolute weight growth of shrimp with hairy eggplant and bitter ginger was higher than the control whiteleg shrimp. This occurs due to the supplementation of hairy eggplant and bitter ginger, which can stimulate the absorption of feed nutrients, mainly due to their protein and bioactive content. Bioactive content such as flavonoids in traditional plants can act as antibacterials and antioxidants, minimizing the increase in pathogenic microflora in the digestive tract. It is thought to increase the digestibility of whiteleg shrimp [33–35].

**Table 1.** The results of the analysis of the production performance parameters of whiteleg shrimp *(L. vannamei).*

Treatments group	Parameter			
	Absolute growth (g)	SGR (%)	FCR	SR (%)
P1	5.82 ± 0.12^a^	5.49 ± 0.04^a^	1.51 ± 0.05^c^	71.92 ± 1.76^a^
P2	6.03 ± 0.06^a^	5.67 ± 0.07^a^	1.45 ± 0.03^c^	76.95 ± 3.03^a^
P3	9.19 ± 0.52^c^	6.69 ± 0.18^c^	0.90 ± 0.08^a^	90.66 ± 4.61^b^
P4	6.97 ± 0.06^b^	5.93 ± 0.03^b^	1.27 ± 0.01^b^	77.09 ± 1.40^a^

SGR: Specific growth rate; FCR: Feed conversion ratio; SR: Survival rate.

The mean ± standard deviation values with different superscript letters show significantly different results (*p* < 0.05).

The best SGR was found in P3 with a growth rate of 6.69%/day, showing that the doses of hairy eggplant and bitter ginger had significant differences (*p* < 0.05) in SGR on whiteleg shrimp. Increased growth rates can occur due to optimal use of feed ingredients. On the other hand, a low growth rate can occur due to health problems, stress, and suboptimal utilization of feed nutrients used for growth [36].

The lowest FCR value was found in P3, then in P2 and P4, respectively. The highest FCR (1.51) was found in the control treatment (Table 1). The FCR was inversely proportional to weight growth, so the lower the FCR, the higher the efficiency of the shrimp in utilizing feed for growth [37]. On the other hand, if the shrimp body is unstable, then the shrimp may experience a decrease in appetite and the feed provided is not converted into biomass. Stressed or unhealthy shrimp can be better at converting feed ingredients for weight growth compared to healthy shrimp [38].

The highest SR was found in P3 (90.66%), and out of all treatments, the lowest percentage was found in the control treatment (71.92%) (Table 1). This indicated that supplementation of hairy eggplant and bitter ginger on whiteleg shrimp had a significant difference in the SR of whiteleg shrimp maintained for 56 days. This is presumably due to the content of secondary metabolites in hairy eggplant and bitter ginger, which can increase the immune system against pathogenic bacterial infections to protect the shrimp body from stress. The biological process will increase due to the involvement of phytochemical substances produced by the extract ingredients, which are capable of producing enzymes for detoxification, modulating the immune system, and increasing shrimp survival. The increased immune response can have a positive impact on increasing body resistance and reducing shrimp mortality. This is confirmed by Jasmanindar et al. [39], who found that the low SR in the treatment without the extract had a relationship with a weak immune system compared to the treatment with seaweed extract. The combination of phytobiotics (thyme essential oil, red thyme, and rosemary pepper) applied in a supplement for 20 days has significant benefits for improving antioxidant protection, reducing the impact of stressors, and modulating the immunity of tilapia against *A. hydrophila* infection [17]*.*

### Water quality

All variables observed in the rearing pond remained within the levels recommended for shrimp culture during the test period (Table 2).

**Table 2.** Water quality parameters during the maintenance period.

Parameter	This study	Optimal range^a^
pH	6.5–8.5	7.5–8.5
Temperature (°C)	27–33	28–30
DO (mg/l)	3.8–4.0	≥ 4
Ammonia (mg/l)	0–0.1	≤ 0.1
Nitrite (mg/l)	0–0.52	≤ 1
Salinity	29.7–33	26–32
Nitrate (mg/l)	0–0.3	0.5

a Ministry of Marine Affairs and Fisheries Regulation (KKP), Republic of Indonesia, 2016.

### Immunity parameters

The hemato-immunological response is a central physiological mechanism, playing a role in protecting animals from disease, environmental stressors, or specific biological agents [40,41], such as phytobiotics. In this study, THC and DHC were carried out to evaluate the immunity condition of whiteleg shrimp with hairy eggplant and bitter ginger during 56 days of rearing. Based on the results, the total hemocytes of whiteleg shrimp in all treatments during the rearing period was around 4.63–16.76 ×10^6^ cells/mm^3^. The highest THC value was found in P3 and was significantly different from the other three treatments (*p* < 0.05). P1 had the lowest THC value of all treatments. P2 and P3 had no significant differences (Table 3).

Hemocytes are the primary mediators of cellular responses in crustaceans, with roles that include self-recognition, phagocytosis, production of reactive oxygen intermediates, wound healing, and the process of melanization by encapsulation of foreign materials [42–44]. The increase in THC of whiteleg shrimp with hairy eggplant and bitter ginger can be suspected due to the effect of bioactive compounds that can modulate shrimp immunity. Other studies confirmed that natural hydrolyzed tannin products from sweet chestnut (*Castanea sativa*) could act as functional feed additives by promoting growth and hematological parameters of whiteleg shrimp [45]. The *in-vivo* effects of Astragalus polysaccharide immunostimulating ingredients, chlorogenic acid, and berberine showed a higher increase in THC in whiteleg shrimp [46].

The difference in THC values in each treatment may be due to the various concentrations of active ingredients in food due to the treatment that has been determined. The low value of THC at P1 (control) may be influenced by physiological factors such as the slow formation of hemocytes in the shrimp body [47,48]. The exciting discussion confirmed that the low number of shrimp hemocytes is due to infiltration of regenerated tissue and hemocyte cell death due to apoptosis [27].

The minor component of shrimp hemocytes in the DHC is hyaline, while the other two types of hemocytes are granular and semi-granular cells. The highest percentage of hyaline was found in P3 (48.67%), followed by P4 (34.67%), P2 (31.33%), and P1 (24.33%) (Table 3). The lowest number of semi-granular cells was found in P3 (23.67%). The decreasing number of semi-granular cells was shown in all treatments, except control (P1), with the highest number (Table 3). The same thing happened to the number of granular cells in the treatment group. The highest number of granular cells was found in P1/control (40.33%).

Hyaline cells have a vital role in the shrimp’s defense system. This cell type has a high ratio of cytoplasmic nuclei and few cytoplasmic granules. An increase in the number of hyaline cells can be associated with phagocytic activity when in contact with antigens or immunostimulating substances that will stimulate the body’s defense activity to evoke the first defense response [49]. Semi-granular cells have a relationship with the addition or reduction of hyaline cells; so, the decrease in the number of semi-granular cells in the treatment group was due to the process of further development into hyaline cells. As a result, these cells cannot develop into semi-granular cells, so the number of semi-granular cells is low. Semi-granular cells are more involved in the encapsulation mechanism. The encapsulation process is a defense reaction against a large number of foreign particles that cannot be phagocytized by hyaline cells. These cells respond more to polysaccharide compounds found in bacterial cell walls.

Granular cells are the type of hemocytic cells that have the most significant size with an active nucleus in the storage process until the release of prophenoloxidase and cytotoxicity. In this study, an increase in granular cells also occurred in the control group (P1). This is due to the low number of hyaline cells involved in the first defense process, thus relying on granular cells for nonspecific body defense, which is driven by the influence of immunostimulatory components such as vitamin C.

### Histology analysis

**Figure 2.**
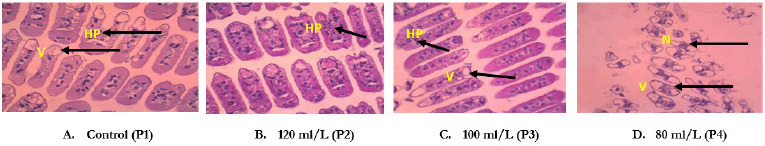
Histology of gill tissue of whiteleg shrimp (L. vannamei). HP: Hyperplasia; V: Vacuolization; N: Necrosis.

The condition of gill tissue on whiteleg shrimp showed that treatments P1 (control) and P3 experienced vacuolation and hyperplasia (Fig. 2), followed by treatments P2, which only experienced hyperplasia, and treatment P4, which experienced vacuolation and necrosis.

The histology can explain that gill tissue damage occurred in all treatments carried out. Physiological differences between each whiteleg shrimp can cause tissue damage even when extracted ingredients are used. More research is needed to prove these results. High hemocytes can indicate infection or stress factors. Clogged blood flow in the lamellae (due to physical trauma, pollutants, or other physiological disturbances) can cause edema (cell swelling) between the blood vessels and the epithelial lining of the primary lamellae. Miller and Zachary [50] explained that necrosis is acute cell damage and can be massive, resulting in incomplete tissue formation due to shrinkage or complete shrinkage of the nucleus. Hyperplasia is the formation of excessive tissue due to an increase in the number of cells so that lamellae with hyperplasia will experience thickening of epithelial tissue at the ends of the filaments or the epithelium located near the base of the gill lamellae [51].

**Table 3.** Immunity parameters (THC and DHC) of whiteleg shrimp with hairy eggplant and bitter ginger.

Treatment Groups	THC (× 10^3^ cells/mm^3^)	Hialin (%)	Semi granular (%)	Granular (%)
P1	4.63 ± 2.05^a^	24.33 ± 2.08^a^	35.33 ± 1.52^c^	40.33 ± 0.57^d^
P2	8.63 ± 0.87^b^	31.33 ± 1.52^b^	31.00 ± 1.00^b^	37.67 ± 0.57^c^
P3	16.76 ± 0.90^c^	48.67 ± 4.04^c^	23.67 ± 3.51^a^	27.67 ± 0.57^a^
P4	9.96 ± 1.75^b^	34.67 ± 1.52^b^	30.33 ± 0.57^b^	35.00 ± 1.73^b^

THC: Total hemocyte counts.

The mean values with different superscript letters show significantly different results (*p* < 0.05).

Based on the results, a 100 ml/l combination of hairy eggplant and bitter ginger in the feed is auspicious for whiteleg shrimp, related to their production performance and immunity. However, further studies are still needed to emphasize the potential that this combination of extracts can provide against pathogenic bacteria and viruses in whiteleg shrimp.

## Conclusion

The supplementation of hairy eggplant and bitter ginger into the feed affected the production performance of whiteleg shrimp as indicated by absolute weight growth, SGR, low FCR, and high survival as shown in P3 (dose of 100 ml/l). Similarly, the immunity parameters, including THC and DHC, were high in the P3 treatment group, although there was a tendency for higher semi-granular and granular cell values in the control group. All these results indicate that the combination of hairy eggplant and bitter ginger as phytobiotic ingredients could act as an exemplary modulator of the nonspecific immune response of whiteleg shrimp, which ultimately increases production performance. Further studies are needed to obtain valuable information regarding the combination of this extract against infection with whiteleg shrimp pathogens, such as viruses and bacteria, to be used as a preventive agent.

## List of Abbreviations

a.m., ante meridiem; °C, degrees Celsius; DO, dissolved oxygen; DHC, differential hemocytes counts; FCR, feed conversion; gm, gram; l, liter; ml, milliliter; µm, micrometer; p.m., post meridiem; SGR, specific growth rate ratio; SR, survival rate; THC, total hemocyte count.
